# Comparative Indoor and Outdoor Degradation of Organic Photovoltaic Cells via Inter-laboratory Collaboration

**DOI:** 10.3390/polym8010001

**Published:** 2015-12-23

**Authors:** Charles Owens, Gretta Mae Ferguson, Martin Hermenau, Eszter Voroshazi, Yulia Galagan, Birger Zimmermann, Roland Rösch, Dechan Angmo, Gerardo Teran-Escobar, Christian Uhrich, Ronn Andriessen, Harald Hoppe, Uli Würfel, Monica Lira-Cantu, Frederik C. Krebs, David M. Tanenbaum

**Affiliations:** 1 Department of Physics & Astronomy, Pomona College, Claremont, CA 91711, USA; cao02010@mymail.pomona.edu (C.O.); gmf02010@mymail.pomona.edu (G.M.F.); 2 Heliatek GmbH, Treidlerstraße 3, 01139 Dresden, Germany; Martin.Hermenau@heliatek.com (M.H.); christian.uhrich@heliatek.com (C.U.); 3 Imec, Kapeldreef 75, 3000 Leuven, Belgium; voroshaz@imec.be; 4 Holst Centre, High Tech Campus 31, 5656 AE Eindhoven, The Netherlands; yulia.galagan@tno.nl (Y.G.); ronn.andriessen@tno.nl (R.A.); 5 Fraunhofer ISE, Heidenhofstrasse 2, D-79110 Freiburg, Germany; Birger.Zimmermann@ise.fraunhofer.de (B.Z.); uli.wuerfel@ise.fraunhofer.de (U.W.); 6 Institut für Physik, Ilmenau University of Technology, Weimarer Str. 32, 98693 Ilmenau, Germany; roland.roesch@tu-ilmenau.de (R.R.); harald.hoppe@tu-ilmenau.de (H.H.); 7 Center for Energy and Environmental Chemistry Jena (CEEC), Friedrich Schiller University Jena, Philosophenweg 7, 07743 Jena, Germany; 8 Laboratory of Organic and Macromolecular Chemistry, Friedrich-Schiller-Universität Jena, Humboldtstr. 10, 07743 Jena, Germany; 9 Department of Energy Conversion and Storage, Technical University of Denmark, DTU, Frederiksborgvej 399, 4000-Roskilde, Denmark; deca@dtu.dk (D.A.); frkr@dtu.dk (F.C.K.); 10 ICN2-Institut Catala de Nanociencia i Nanotecnologia, Campus UAB, 08193 Bellaterra (Barcelona), Spain; gerardo.teran@cin2.es (T.T.-E.); monica.lira@cin2.es (M.L.-C.); 11 CSIC-Consejo Superior de Investigaciones Cientifcas, ICN2 Building Campus UAB, 08193 Bellaterra (Barcelona), Spain

**Keywords:** degradation effects, environmental degradation, organic photovoltaic cells, outdoor testing, polymer photovoltaic cells, small molecule photovoltaic cells, power conversion efficiency, stability

## Abstract

We report on the degradation of organic photovoltaic (OPV) cells in both indoor and outdoor environments. Eight different research groups contributed state of the art OPV cells to be studied at Pomona College. Power conversion efficiency and fill factor were determined from IV curves collected at regular intervals over six to eight months. Similarly prepared devices were measured indoors, outdoors, and after dark storage. Device architectures are compared. Cells kept indoors performed better than outdoors due to the lack of temperature and humidity extremes. Encapsulated cells performed better due to the minimal oxidation. Some devices showed steady aging but many failed catastrophically due to corrosion of electrodes not active device layers. Degradation of cells kept in dark storage was minimal over periods up to one year.

## 1. Introduction

During the 1980s, the first polymer cells, such as polythiazyl, were researched in photovoltaic cells. Current polymer organic photovoltaic (OPV) cells have been demonstrated with efficiencies over 11% [1]. Small-molecule cells while distinctly different from polymer OPVs have seen similar progress also demonstrating efficiencies of 12%, and impressive stability [2]. A successful PV technology must be able to demonstrate a balance of three main attributes: efficiency, cost, and lifetime. OPV technologies are remarkable in their demonstrated potential for low cost manufacturing and modest embedded energy [3]. The demonstration of power conversion efficiencies (PCEs) over 10% and continuously improving in research cells from a variety of materials systems is a major achievement [4]. The issue of cell lifetime and an understanding of cell degradation have also been addressed in some laboratory studies [5,6,7,8,9,10,11,12], and a few outdoor tests [13,14,15]. Of the three major attributes needed, lifetime in realistic outdoor settings is probably the most significant challenge left to address. Most importantly, all three attributes must be solved together in a single module to be successful.

In this study, we report on the performance and degradation of a wide variety of OPV cells in both indoor and outdoor environments. Eight different research groups contributed state of the art OPV cells to be studied by a single lab (Pomona College) under standardized conditions in southern California. Power conversion efficiency, fill factor, and other parameters are extracted from IV curves collected at regular intervals for each cell over the course of six to eight months. Sets of similarly prepared devices were measured indoors, outdoors, and after dark storage. Different device architectures are represented for each of the eight laboratories and some laboratories prepared multiple variations of the devices. In most cases, large numbers of nominally identical OPV devices were provided to ensure consistency and redundancy in the experimental trials.

The cells placed outdoors were measured in accordance with the ISOS-O-2 Outdoor standards. Indoor cells were measured in accordance with the ISOS-L-2 Laboratory standards [16]. In addition, a series of cells were placed in dark storage for a one-year period and characterized at the end of the study in same test bed as the indoor cells.

## 2. Results

**Figure 1:**
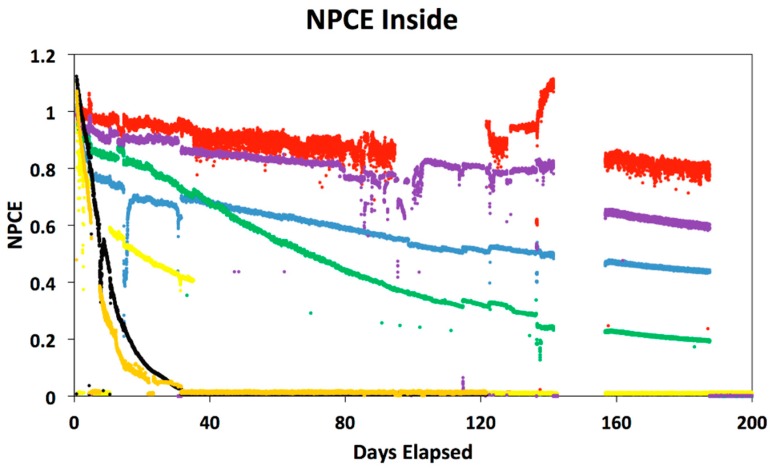
Graph displaying the indoor normalized power conversion efficiency (NPCE) curves for cells from Groups 1–7. Colors correspond to labels in Table 1.

**Figure 2:**
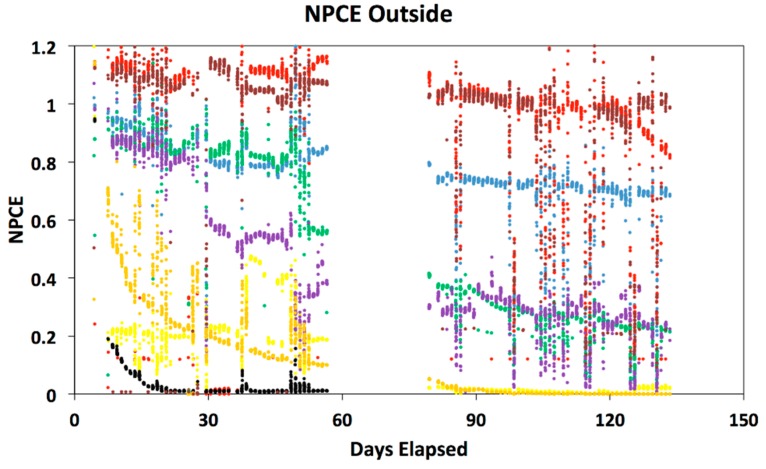
Graph displaying the outdoor normalized power conversion efficiency (NPCE) curves for each cell group. Colors correspond to labels in Table 1.

Table 1 displays the initial PCE values used as normalization factors used in calculating the normalized power conversion efficiency of the cells, as well as estimates for T80, T50, and T End, the number of days to decay to 80%, 50% or failure of the cell or contacts. Figure 1 and Figure 2 display the normalized power conversion efficiency of the OPV cells in indoor and outdoor settings, respectively. For clarity, cells with the smoothest decay curves are shown. Some cells had catastrophic contact failures (Group 4 is an example.) For the outdoor data in Figure 2, we have only plotted the data collected near mid-day to highlight the long-term stability pattern over the daily oscillations. In both plots, there is a gap in the data where automated collection was interrupted, but the trends are clear. It should be noted that the Group 6 cells had the encapsulation removed, resulting in a rapid degradation in power conversion efficiency. Group 6 cells with the encapsulation were tested and displayed less degradation, although they still suffered from corrosion of the metal contacts. The other cell group that experienced this type of rapid degradation was Group 7, which is unusual since this cell type was encapsulated on glass substrates sealed with a UV curable polymer (Ormocer, from Micro resist technology GmbH, Berlin, Germany). This may indicate that the encapsulation either failed or did not provide a suitable barrier against the humidity and oxygen. This failure in encapsulation may be due to cracking the encapsulation during the mounting process before data collection or during the time the cells were in transit from their home location.

**Table 1:** Characterization of cells in study.

Cell type	Location	Initial PCE (%)	T80 (days)	T50 (days)	T End (days)
●Group 1	Outdoor	3.23	30	>130	>130
	Indoor	5.22	5	140	>190
●Group 2	Outdoor	10.41	>130	>130	>130
	Indoor	11.84	175	>190	>190
●Group 3	Outdoor	1.83	43	~66	>130
	Indoor	3.07	23	72	>190
●Group 4	Outdoor	4.20	<7	<7	85
	Indoor	7.77	2	20	37
●Group 5	Outdoor	2.62	26	49	>130
	Indoor	6.01	79	>190	>190
●Group 6	Outdoor	1.98	<7	<7	20
	Indoor	1.60	5	9	36
●Group 7	Outdoor	1.45	<7	9	88
	Indoor	2.72	4	6	32
●Group 8	Outdoor	2.23	>130	>130	>130

## 3. Discussion

While there is certainly more noise in the measurements of the outdoor cells, the trends in the outdoor and indoor data very similar. The Group 2 small molecule cells are the most stable in both cases. In fact, most of the cells show similar stability in both indoor and outdoor measurements, with two exceptions. The Group 5 cell mounted outside decayed significantly more rapidly in comparison to the corresponding indoor cell.

The shapes of the decay curves are not uniform among the cells, with some cells showing a nearly linear decay, while others show rapid exponential decay. On the time scale of 100 days, Groups 1, 2, and 8 are impressive even in the outdoor environment, comparable to the indoor performance, suggesting the encapsulation methods are effective. Over longer time scales ~200 days, most of the outdoor cells showed catastrophic failures. Visual inspection of these cells suggests that metal thin film contact electrodes become corroded at this point, distinctly different from the gradual degradation shown here.

After one year in climate controlled dark storage, cells from Groups 1–7 were characterized again. All these cells were still functional, with Groups 5 and 7 showing over 50% decay in PCE, but most cells showed less than 30% degradation, and Group 3 cells even demonstrated a modest improvement.

## 4. Materials and Methods

**Figure 3:**
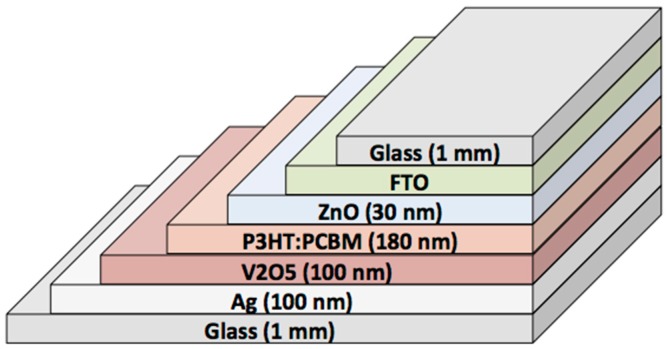
A schematic diagram shows the layers of the Group 7 devices where the P3HT:PCBM is the photo-active layer, ZnO is the ETL, V_2_O_5_ is the HTL, Fluorine Tin Oxide is a transparent cathode, and Ag is the anode. The device is deposited on and encapsulated by glass using a UV curing polymer.

The following information details the chemical makeup and construction of each cell type used in this study. In order to maintain objectivity in the analysis process, the cell type names are kept anonymous throughout the paper and referred to simply as Group 1 through Group 8. All cells were fabricated and mailed to Pomona College for evaluation in this study. A layer-by-layer description of each cell type is seen in Table 2. While the details are distinct, the structures primarily consist of a photo-active bulk hetero-junction layer where photons are absorbed to create excitons sandwiched by a hole transport layer (HTL) and an electron transport layer (ETL), which is again sandwiched by an anode and cathode. Either the anode and HTL or the cathode and ETL must be transparent to enable photons to reach the photoactive layer. Figure 3 shows a schematic representation of the layers in the Group 7 cell structure. Different photoactive layers can be used to harvest different portions of the solar spectrum and devices with multiple stacked junctions (Group 2) can have significantly higher efficiencies although the fabrication processes are more complex. Four of the device structures in this study use P3HT:PCBM for the photo-active layer, while two use PCDTBT:PCBM, and two use small molecules deposited via thermal evaporation in vacuum. Group 8 devices are fabricated in ambient from liquid precursors in a roll-to-roll fabrication scheme on PET foils, while the others are on glass substrates. In some devices (not used in this study), both sides are transparent in some portion of the visible spectrum enabling the devices to be considered for window treatments that can reduce direct sun while simultaneously generating electrical power.

**Table 2:** Summary of Cells in Study.

Identifier	Cell stack layers [thickness]	Encapsulated	Encapsulation scheme
Group 1	Cr [5 nm] + Al [100 nm] + Cr [5 nm] + P3HT:PCBM [220 nm] + PEDOT:PSS [200 nm] + Au grid [100 nm]	27/1/2013	Glass substrates sealed with Delo-Katiobond (LP655)
Group 2	TCO + *N*-Doped OrganicMix1 + OrganicMix1 + *P*-Doped OrganicMix1 + *N*-Doped OrganicMix2 + OrganicMix2 + *P*-Doped OrganicMix2 + Metal	18/1/2013	Glass substrate sealed with an unspecified epoxy glue
Group 3	ITO [120 nm] + ZnO [30 nm] + P3HT:PCBM [240 nm] + HTL + Ag + Metal Lid	4/2/2013	Metal Lid attached to glass substrate using Huntsman Araldte 2014-1
Group 4	ITO [120 nm] + ZnO[~30 nm] + P3HT:PCBM [80 nm] + MoO_3_ [10 nm] + Ag [150 nm] + Al [150 nm]	14/1/2013	Glass substrates sealed with an unspecified UV curable glue
Group 5	ITO + PEDOT:PSS + PCDTBT:PCBM + TiO_2_ + Al	17/12/2012	Glass substrates sealed with an unspecified UV curable glue
Group 6	AL [100 nm] + Bphen [6 nm] + C_60_ [30 nm] +ZnPc:C_60_ [30 nm] + DF-DPB:C_60_F_36_ [30 nm] + C_60_F_36_ [1 nm] + ITO	14/1/2013	Glass substrates sealed with a UV curable epoxy (Nagase) and a getter sheet (Dynic Ltd.)
Group 7	FTO + ZnO [50 nm] + P3HT:PCBM [30 nm] + V_2_O_5_ [100 nm] + Ag [100 nm]	23/1/2013	Glass substrates sealed with a UV curable polymer, Ormocer from Micro resist technology GmbH
Group 8	PET + Ag + PEDOT:PSS + ZnO + P3HT:PCBM [400 nm] + PEDOT:PSS + Ag + PET	24/9/2012	PET foils with barrier properties (0.01 cm^3^/(m^2^·bar·day) for oxygen and 0.04 g/(m^2^·day) for water vapor) and a UV cut-off (390 nm).were laminated together using Delo epoxy.

**Figure 4:**
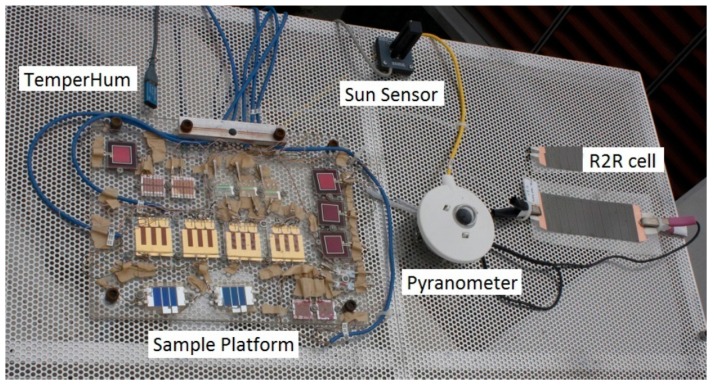
Cells used in this study mounted on the outdoor solar tracking platform along with the pyranometer, sun tracking sensor, and temperature and humidity sensors.

Upon receipt of cells from the collaborators, 2 layers of silver paint were applied to all evaporated metal electrodes in order to increase their resilience. Preliminary IV characterization, were performed for each cell using a light emitting plasma (LEP) lamp. After the initial data were analyzed, 3 test cells from each cell type were chosen based on having similar electrical properties and high performance capabilities. Two cells from this batch were measured on the outdoor rooftop solar tracker while the last sample was measured on the indoor laboratory system. Remaining cells were placed in dark storage. Electrical contacts were made with a variety of mini alligator clips that were selected based on their fit to the specific cell contact geometry. Indoor samples were placed uniformly around the LEP lamp and held in place with adhesive tape. Outdoor samples were mounted with screws and washers to an insulating base plate and mounted on the solar tracker with measurement cables stress relieved to the base plate. Figure 4 shows the 8 types of devices mounted for the outdoor measurements.

A solar tracker was employed on the roof a building in southern California to measure the performance of the OSCs in an outdoor environment in which they are always facing the sun. This allows for the maximum harvesting efficiency.

A dual axis sun sensor, containing photodiodes (Heliotrack, LLC, Bellvue, CO, USA) was used to align the tracker with the sun. The photodiodes are arranged along the central vein of the shadow box’s four vertical walls to track alignment. Stepper motors provide dual axis motion. Controlling the entire setup was an Arduino microcontroller (Adafruit, New York, NY, USA).

Degradation of cells on the tracker can come about due to the light intensity, temperature, and humidity of the outdoors. Irradiance was measured with a pyranometer (Kipp and Zonen, Delft, The Netherlands). In order to monitor the temperature and humidity, a sensor (TEMPerHum by RDing Technology, Shenzhen, China) was used. The sensor measured the temperatures within 2 °C and the humidity within 5% RH at discrete time intervals, and the sensor data were logged along with the irradiance and cell data on a laptop computer. Overall the outdoor conditions saw humidity levels from 5% to 95% and temperatures from 10 to 45 °C during the experiments, while the indoor conditions were much more stable with humidity below 30% and temperatures between 30 and 50 °C.

The LEP lamp (Chameleon Plasma Grow Lighting, Ocoee, FL, USA) was used in the indoor setup in order to simulate sunlight. The lamp provided an irradiance of 50 mW/cm^2^ on the cells continuously and has a good match for the solar spectrum at wavelengths from 400–600 nm. A silicon control cell was used to monitor the lamp stability. A pyranometer verified the irradiance equivalent to half a sun. Cells were connected to the data acquisition system and a sensor monitored the temperature and humidity.

A SourceMeter (Keithley, Cleveland, OH, USA) combined with a multiplexing relay performed IV sweeps while software (LabVIEW, National Instruments, Austin, TX, USA) switched between 20 different devices, recording data and logging the files. Similar setups existed for both the indoor and the outdoor measurements. Cells were measured continuously on 10 or 15 min intervals and were left open circuit between measurements. Full IV curves are stored along with key parameters and summary data for each measurement cycle.

## 5. Conclusions

There has been significant progress on the development of organic photovoltaic devices. A wide variety of device architectures are employed in this study including different photoactive layers, different electron and hole transport layers, different electrode materials, and different encapsulation techniques. No single laboratory is likely to develop the expertise to work with all theses different approaches in house and thus such a broad comparison is only possible with an inter-laboratory approach. While each home laboratory has its own characterization facilities and protocols, the standardization of characterization schemes remains a challenge in this research field where subtle differences in equipment and protocol have been known to have significant influence upon measured characteristics. By agreeing as a community to fabricate and share devices to be tested concurrently at a single site with a uniform multichannel characterization apparatus, comparisons of different device architectures are far more likely to provide meaningful results. Inter-laboratory collaborations with a single characterization site are the only way to make reliable comparisons between devices fabricated at independent laboratories for outdoor studies where irradiance, humidity and temperature are all highly variable. Real world outdoor testing in this study demonstrates the benefits of robust encapsulation techniques. We observe that the best devices were impressive and highly stable until the failure of exposed metal thin film electrodes occurred due to corrosion. Simultaneous indoor studies were seen to be good predictors of outdoor performance in most cases, suggesting that significant progress with encapsulation technology has been achieved in these devices. The corrosion and failure of thin film metal contacts outside of the encapsulation is clearly observed in our study and can be addressed in future studies, as has been demonstrated in the commercialization of more mature PV technologies. However, the modest degradation of devices kept in dark climate controlled storage over a one year period are not likely due to electrode corrosion and suggest that there are still degradation pathways that need to be explored further with analytical techniques beyond electronic IV curve measurements.
